# Targeted Acne Therapy Using Light-Absorbing Gold Microparticles Combined With Long-Pulsed 1064 nm Nd:YAG Laser: Case Series

**DOI:** 10.7759/cureus.98929

**Published:** 2025-12-10

**Authors:** Ruri Pamela, Maria Sutanto, Andrea Christanty, Ervina Ervina, Haewoong Lee

**Affiliations:** 1 Department of Dermatology, Venereology, and Aesthetic, General Soedirman National Defense Central Hospital, Jakarta, IDN; 2 Department of Dermatology, Louis Dermatologic Clinic, Guri, KOR

**Keywords:** acne vulgaris knowledge, acne vulgaris therapy, gold microparticles, inflammatory acne vulgaris, photothermal therapy

## Abstract

Acne vulgaris is a prevalent dermatological disorder associated with inflammation and sebaceous gland hyperactivity. Conventional therapies, including topical and systemic agents, are limited by side effects, antimicrobial resistance, and frequent relapse. Light-absorbing gold microparticles activated by long-pulsed 1064 nm Nd:YAG laser therapy represent a novel, non-invasive approach targeting sebaceous glands. However, clinical data remain limited, particularly regarding optimal dosing. We report a series of four patients aged 19-31 years with moderate to severe acne vulgaris. Two patients received a low dose (1-2 cc), and two received a standard dose (2.5-3.5 cc) of gold microparticle suspension applied with sonophoresis, followed by 1500 shots of long-pulsed 1064 nm Nd:YAG laser. Each underwent three treatment sessions at two-week intervals. Clinical outcomes were assessed using standardized photography, Janus skin analyzer evaluation, and patient-reported satisfaction two weeks after the final session. Patients treated with the standard dose demonstrated marked reductions in inflammatory lesions, post-acne erythema, and skin oiliness, with high satisfaction and no recurrence during follow-up. In contrast, patients receiving the low dose showed only moderate improvement, with partial reduction in lesion counts and erythema. No adverse effects or complications were observed across all cases. This case series suggests that higher doses of gold microparticles combined with long-pulsed 1064 nm Nd:YAG laser therapy may provide superior clinical outcomes compared to lower doses in the management of moderate to severe acne vulgaris. Nonetheless, treatment with either dose was safe, well-tolerated, and associated with moderate-to-high patient satisfaction. Although encouraging, these findings are limited by a small sample size, and larger controlled studies with extended follow-up are needed to confirm efficacy and optimize treatment protocols.

## Introduction

Acne vulgaris is one of the most prevalent dermatological disorders, affecting up to 9.4% of the global population [1]. It is characterized by pilosebaceous unit pathologies, involving follicular hyperkeratinization, excess sebum production, inflammation, and specific bacterial skin colonization [2]. Conventional therapies, including topical retinoids, benzoyl peroxide, oral antibiotics, hormonal agents, and oral isotretinoin, remain the mainstay of therapy. However, these are often limited by side effects, antimicrobial resistance, and poor adherence [3]. These challenges have encouraged exploration of alternative physical modalities, particularly laser- and light-based treatments [4].

The neodymium-doped yttrium aluminum garnet (Nd:YAG) laser has demonstrated efficacy in acne management by targeting vascular elements of inflammatory lesions and inducing thermal injury to sebaceous glands, thereby reducing sebum production [5-7]. However, its selectivity is hindered by competing absorption from endogenous chromophores such as water, melanin, and hemoglobin [8].

To overcome this limitation, the use of exogenous light-absorbing agents has been proposed [9]. Gold microparticles, with strong optical absorption in the near-infrared spectrum, preferentially localize within sebaceous follicles and enhance selective photothermolysis when activated by Nd:YAG irradiation [10]. Preliminary studies suggest that this combined approach effectively destroyed sebaceous glands [11].

Preclinical and early clinical studies have reported encouraging outcomes using gold microparticles in combination with ND:Yag laser therapy, but evidence and clinical applications remain limited. In particular, there is little published data describing practical application, dosage considerations, and real-world patient outcomes.

Herein, we present a case series of four patients with moderate to severe acne vulgaris treated with light-absorbing gold microparticles combined with long-pulsed 1064-nm Nd:YAG laser therapy. This report aims to highlight the clinical response, safety, and feasibility of this novel therapeutic approach’s role in acne management.

## Case presentation

Patients and study design

This report describes a prospective case series conducted at General Soedirman National Defense Central Hospital (Jakarta, Indonesia) between April and June 2024. A total of four patients with moderate to severe acne vulgaris were included. All patients were informed in detail about the nature of the treatment, expected benefits, and possible risks. Written informed consent for treatment and publication was obtained from all participants prior to enrollment.

Eligible patients were males and females aged 16-35 years with clinically diagnosed moderate to severe acne vulgaris (based on the Global Acne Severity Scale [12]) affecting the face, who had not received any topical or systemic acne therapy, laser resurfacing, chemical peeling, or similar interventions for at least three months prior to, during, and one month following the treatment sessions. Exclusion criteria included patients with mild comedonal acne, very severe nodulocystic acne, pregnancy, lactation, active skin infections, any systemic illness interfering with wound healing, allergies to metal or gold, or inability to comply with treatment and follow-up.

Patients were treated with gold microparticle suspension (Simfle Stick-Gold PTT, South Korea) and were blinded to the administered dose. The suspension was applied to affected facial areas and enhanced with sonophoresis at 40 kHz power output to facilitate follicular penetration. This was followed by irradiation with a long-pulsed 1064-nm Nd:YAG laser. Each patient underwent three treatment sessions at two-week intervals. During the intervention period, patients were advised not to use any topical agents, except over-the-counter face wash. Clinical evaluation was performed by the same independent doctor before the first treatment and two weeks following the final session. Standardized digital photographs (frontal, right, and left facial views) of the patients’ skin condition were analyzed using the JANUS Pro skin analyzer (PIE Co. Ltd., South Korea).

Treatment efficacy was assessed by reduction in inflammatory acne lesions, improvement in post-acne erythema, changes in skin oiliness, and patient-reported satisfaction. Patients rated their satisfaction on a three-point scale: (i) full satisfaction, (ii) partial satisfaction, or (iii) no satisfaction. Adverse events, the presence of new acne lesions, and the recurrence of acne lesions were also documented.

Case 1

**Figure 1:**
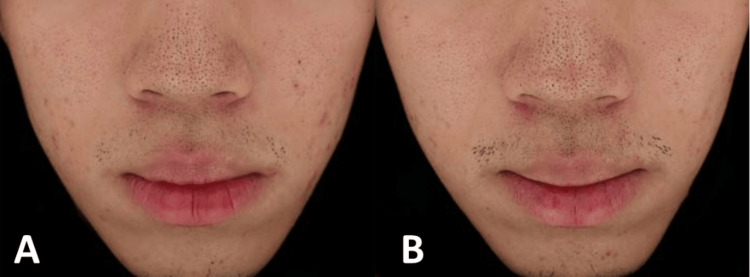
19-year-old male with moderate acne vulgaris: (A) at baseline and (B) two weeks after the last treatment session with gold microparticles and a long-pulsed 1064 nm Nd:YAG laser

A 19-year-old male presented with moderate acne vulgaris involving the cheeks and forehead. He had a history of unsuccessful treatment with topical retinoids and oral antibiotics, discontinued six months before presentation. At baseline, numerous open comedones on the nose and inflammatory papules on the forehead and cheeks were observed. Several ice-pick-shaped atrophic scars were also present on both cheeks (Figure 1A).

Treatment consisted of the application of 1.5 cc of gold microparticle suspension with sonophoresis, followed by 1500 shots of long-pulsed 1064-nm Nd:YAG laser therapy (fluence 4 J/cm², spot 8 mm, pulse duration 0.3 ms). Three sessions were performed at two-week intervals. Two weeks after the final session, the patient demonstrated a moderate reduction in inflammatory lesions and post-acne erythema, notably in the left cheek (Figure 1B). Sebum production, subjectively reported as “greasy skin,” improved slightly. No new lesions were observed during the follow-up. However, no apparent improvement was observed in the open comedones. Overall, the patient reported partial satisfaction with the treatment result and the absence of adverse effects.

Case 2

**Figure 2:**
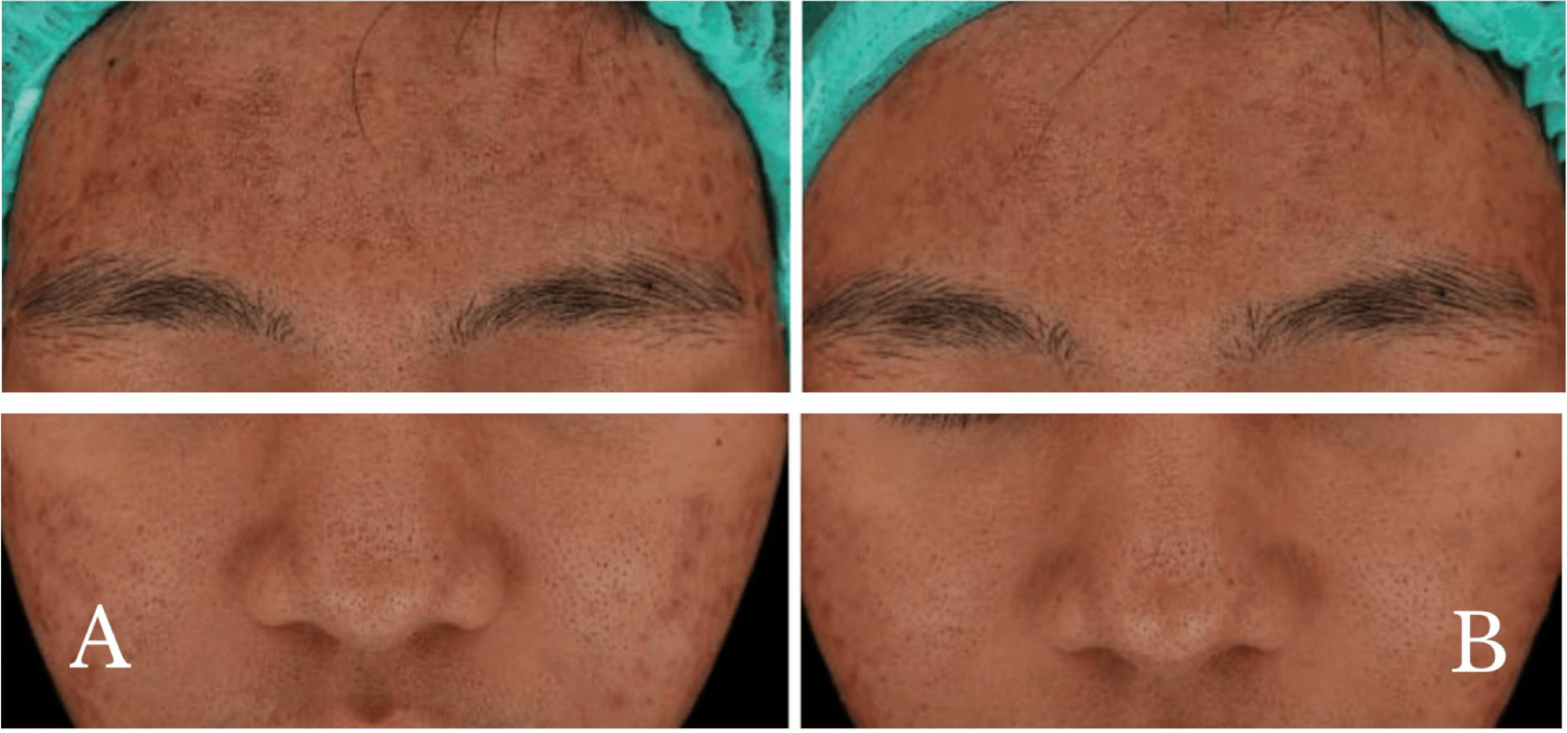
26-year-old female with severe acne vulgaris: (A) at baseline and (B) two weeks after the last treatment session with gold microparticles and a long-pulsed 1064 nm Nd:YAG laser

A 26-year-old female with severe inflammatory acne vulgaris involving the forehead and cheeks was included. She had previously received isotretinoin but discontinued due to poor adherence. At presentation, multiple papulopustules, open comedones, and post-inflammatory hyperpigmented macules were present, most notably on the forehead and cheeks (Figure 2A).

She received 2 cc of gold microparticle solution with sonophoresis, followed by 1500 shots of long-pulsed Nd:YAG laser therapy (fluence 4 J/cm², spot 8 mm, pulse duration 0.3 ms), repeated for three sessions with a two-week interval. At follow-up, there was a significant reduction in inflammatory papules and pustules and noticeable improvement in erythema and hyperpigmentation (Figure 2B). Oiliness of the skin improved modestly, and no new lesion was observed. The patient tolerated treatment well without side effects and was fully satisfied with the treatment.

Case 3

**Figure 3:**
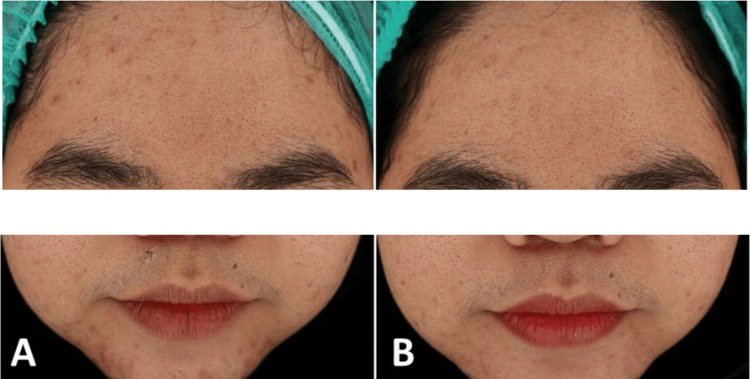
27-year-old female with moderate acne vulgaris: (A) at baseline and (B) two weeks after the last treatment session with gold microparticles and a long-pulsed 1064 nm Nd:YAG laser

A 27-year-old overweight female presented with moderate acne vulgaris localized to the forehead and chin. She had no recent acne treatment and denied any systemic therapy within the past year. Examination revealed multiple inflammatory papules, pustules, and open comedones, without any nodules. Notice that hirsutism was also observed in the patient (Figure 3A).

She underwent treatment with 2.5 cc of gold microparticle suspension, delivered via sonophoresis, followed by 1500 shots of long-pulsed Nd:YAG laser (fluence 4 J/cm², spot 8 mm, pulse duration 0.3 ms). Three sessions were performed at two-week intervals. After treatment, the patient showed a marked reduction in inflammatory lesions and erythema, with near-complete resolution of pustules on the chin (Figure 3B). She also reported significant improvement in skin oiliness and texture. Follow-up showed no recurrence, and patient satisfaction was high with the treatment.

Case 4

**Figure 4:**
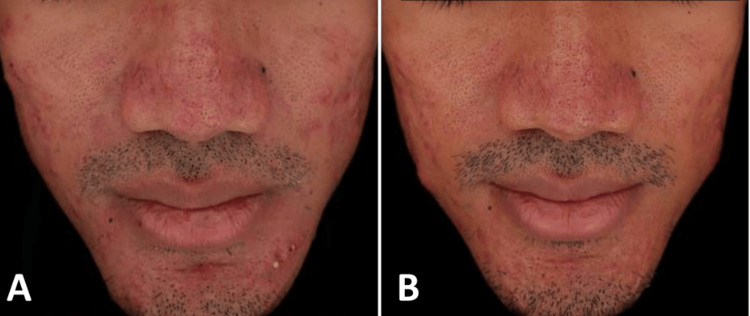
31-year-old male with severe acne vulgaris: (A) at baseline and (B) two weeks after the last treatment session with gold microparticles and a long-pulsed 1064 nm Nd:YAG laser

A 31-year-old male with persistent severe acne presented with numerous open comedones, atrophic scars, extensive papulopustular lesions, and several erythematous nodules on the cheeks, chin, and forehead (Figure 4A). He had previously tried using various topical agents without significant improvement over the last year.

He was treated with 3.5 cc of gold microparticle solution and 1500 shots of long-pulsed Nd:YAG laser (fluence 4 J/cm², spot 8 mm, pulse duration 0.3 ms) in three sessions spaced two weeks apart. By the end of follow-up, the patient exhibited a dramatic reduction in inflammatory lesions and almost complete resolution of erythema, although several papular lesions remained (Figure 4B). No new lesions or relapses were observed. The patient rated his satisfaction as “excellent,” and no adverse events were reported.

## Discussion

Acne vulgaris (AV) remains a highly prevalent dermatological disorder with multifactorial etiology, including sebaceous gland hyperactivity, follicular keratinization, bacterial colonization, and inflammation [1,2]. While conventional topical and systemic treatments have proven effective, their limitations are well recognized [3]. Complex regimens, antibiotic resistance, systemic side effects, and poor patient adherence often compromise long-term outcomes. As a result, energy-based devices have gained attention as alternatives or adjuncts to standard therapy. Among these, selective photothermolysis of sebaceous glands represents an innovative and targeted strategy that addresses the underlying pathophysiology of AV by targeting hyperactive sebaceous glands [4].

Recently, photothermal therapies (PTT) have been developed to induce localized thermal damage to sebaceous glands. Infrared lasers and radiofrequency devices demonstrated partial efficacy but lacked specificity, mainly in pigmented skin, with risks of scarring and dyspigmentation [5,8]. Selective photothermolysis, facilitated by exogenous chromophores, has provided a breakthrough toward more precise sebaceous gland targeting [9].

Gold nanoparticles and microparticles have emerged as highly effective exogenous chromophores due to their favorable optical properties, biocompatibility, minimal toxicity, and efficient conversion of near-infrared light into localized heat [9]. Moreover, when activated by 800-1064 nm lasers, these particles accumulate within sebaceous glands and hair follicles, allowing for selective glandular destruction while sparing surrounding tissues. Randomized trials by Paithankar et al. using gold-coated silica microparticles with 800 nm diode lasers for moderate-to-severe facial acne reported significant reductions in inflammatory lesions compared to controls after six weeks [10]. Similarly, Waibel et al. demonstrated significant reductions in inflammatory lesion counts with gold microparticles and 1064-nm Nd:YAG laser therapy combined with topical retinoid pre-treatment in 52 subjects with mild-to-moderate facial acne. This method achieves up to 68% reduction of inflammatory lesion counts at 12 months, alongside high patient satisfaction and tolerability [13]. Further experimental study using porcine skin demonstrated that application of a mixture of gold and platinum nanoparticles by a combination of microchanneling and sonophoresis, followed by a 1064-nm long-pulsed high-fluence Nd:YAG laser, effectively destroyed the sebaceous follicles [11].

Despite these promising results, variability persists regarding optimal gold microparticle volume, delivery method, and laser parameters. Our case series adds to the available evidence by directly comparing clinical outcomes between patients treated with low-dose (1-2 cc) and standard-dose (2.5-3.5 cc) gold microparticles combined with a long-pulsed 1064 nm Nd:YAG laser. We observed more pronounced improvements in inflammatory lesion clearance, erythema reduction, and sebum control in patients receiving the higher dose, whereas those treated with lower doses achieved only moderate improvement.

Importantly, no recurrences or significant adverse effects were reported, indicating the safety and tolerability of this modality. The therapeutic effect of gold microparticles combined with a long-pulsed 1064 nm Nd:YAG laser on acne lesions is achieved through selective photothermolysis of blood vessels, possibly by increasing secretion of TGF-β, decreasing IL-8 levels, and inducing thermal damage to sebaceous glands. Moreover, the advantage of using the 1064-nm wavelength is that it provides deeper tissue penetration and is less susceptible to melanin absorption [5,9]. Together, these effects probably contribute to a reduction in sebum production, which is key in AV pathogenesis.

Our findings corroborate existing evidence supporting nanoparticle-assisted laser therapy as an effective and safe treatment for moderate-to-severe AV. By demonstrating that higher doses achieve superior outcomes without additional risks, this case series strengthens the rationale for standardizing dosing protocols in clinical practice. Moreover, these results reinforce the evidence of selective photothermolysis as a novel approach in acne management. Unlike conventional therapies, which primarily modulate inflammation or microbial load, nanoparticle-assisted photothermal therapy directly targets sebaceous gland hyperactivity, thereby addressing a root driver of acne pathogenesis.

## Conclusions

This case series highlights the potential dose-dependent efficacy of gold microparticles combined with long-pulsed 1064 nm Nd:YAG laser therapy in moderate to severe acne vulgaris. While promising, the strength of evidence provided by this case series is inherently limited by its small sample size, lack of randomization, and absence of long-term follow-up. In addition, as an uncontrolled study, causality between microparticle dose and clinical outcomes cannot be firmly established. Furthermore, the subjective nature of patient-reported improvement and the absence of standardized lesion counting scales limit the generalizability of findings. Larger randomized controlled trials with extended follow-up are needed to validate these observations, refine dosing strategies, and further clarify the safety profile.
